# Deguelin induces the apoptosis of lung cancer cells through regulating a ROS driven Akt pathway

**DOI:** 10.1186/s12935-015-0166-4

**Published:** 2015-02-25

**Authors:** Huae Xu, Xiaolin Li, Wenqiu Ding, Xiaoning Zeng, Hui Kong, Hong Wang, Weiping Xie

**Affiliations:** Department of Pharmacy, The First Affiliated Hospital of Nanjing Medical University, Nanjing, People’s Republic of China; Department of Respiratory Medicine, The First Affiliated Hospital of Nanjing Medical University, 300# Guangzhou Road, Nanjing, 210029 People’s Republic of China; Department of Geriatric Gastroenterology, The First Affiliated Hospital of Nanjing Medical University, Nanjing, People’s Republic of China

**Keywords:** Deguelin, Lung cancer, ROS, Akt, Apoptosis

## Abstract

**Background:**

Duguelin is a rotenoid extracted from plants and has potent antitumor effects in vitro and in vivo. However, the mechanism underlying the antitumor effect remains unclear. Our preliminary study showed that Deguelin is effective to stimulate the generation of Reactive Oxygen Species (ROS). In the current study, we evaluated the in vitro cytotoxicity of Deguelin against lung cancer cells and studied whether a ROS scavenger, N-acetyl-cysteine (NAC), can reverse the inhibitory effect of Deguelin.

**Results:**

We showed that the dose-dependent apoptotic inducing effect of Deguelin could be partially reversed by the co-administration of NAC. Moreover, Deguelin reduced the phosphorylation of Akt protein and induced the apoptotic protein Caspase-3 in a dose-dependent manner. Co-treatment with NAC partially attenuated this effect and rescued some cells from apoptosis.

**Conclusion:**

Deguelin induces the apoptosis of cancer cells through a ROS driven Akt pathway, which could translate into a promising therapeutic for lung cancer.

## Background

Lung cancer remains the leading cause of cancer related death all over the world [1]. However, traditional chemotherapy treatments often lead to severe toxicity and may encounter the emerging resistance [2]. Therefore, it is urgent to identify alternative drugs to chemotherapeutics with lower toxicity and substantial antitumor effect.

Deguelin, a rotenoid extracted from the African plant *Mundulea sericea*, has been reported to inhibit the *in vitro* and *in vivo* growth of several types of tumors, including lung, gastric, breast, colon and rectal cancer etc. [3-7]. The possible mechanism may involve DNA damage and repair gene suppression and regulation of tumor specific pathways [5,6,8]. Additionally, Deguelin has been demonstrated to induce the apoptosis of tumor cells by inactivating Akt phosphorylation [9,10]. As a survival signal, activation of the Akt pathway renders cells resistant to apoptosis through regulation of pro-apoptotic and anti-apoptotic proteins [11-13]. Previous studies have shown that targeting the Akt pathway effectively inhibits the proliferation of cancer cells and significantly delays tumor growth [12,13].

Reactive oxygen species (ROS), a natural byproduct of the normal metabolism of oxygen plays a significant role in a number of normal biochemical functions and abnormal pathological processes [14]. There is a subtle balance between intracellular ROS and antioxidant capacity in normal cells, which determines their destiny [15]. Yet cancer cells are characterized of elevated intrinsic ROS stress, resulting from carcinogenesis stimulation, abnormal metabolic activity, and mitochondrial malfunction [16,17]. The limited capacity of the tumor cells to deal with the elevating ROS levels makes them vulnerable to oxidative stress [16]. This embarks a novel strategy for cancer therapy of promoting apoptosis of cancer cells by eliminating the ability of antioxidant defense systems through inducing ROS production [18,19].

In our preliminary studies, we showed that Deguelin affects the redox state by inducing intracellular reactive oxygen species (ROS) generation. Moreover, there is substantial evidence showing that ROS is an upstream signal of the Akt pathway [20-22]. The effect of both Akt pathway inhibition and oxidative stress combine to induce destruction of cancer cells [23-26]. There is no detailed report, however, about the influence of Deguelin on intracellular ROS earlier in paper since ROS is considered as an upstream signal to the Akt pathway. We hypothesized that Deguelin may induce cellular apoptosis through induction of ROS, which may suppress the downstream Akt pathway contributing to the cell death.

Here we investigated the antitumor effect of Deguelin and the underlying mechanisms in non small cell lung cancer cell line NCI-1975. Our results strongly suggest that ROS-driven Akt dephosphorylation was vital to the antitumor effect of Deguelin, which could be partially reversed by an antioxidant NAC.

## Results

### Elevation of intracellular ROS levels by Deguelin

**Figure 1:**
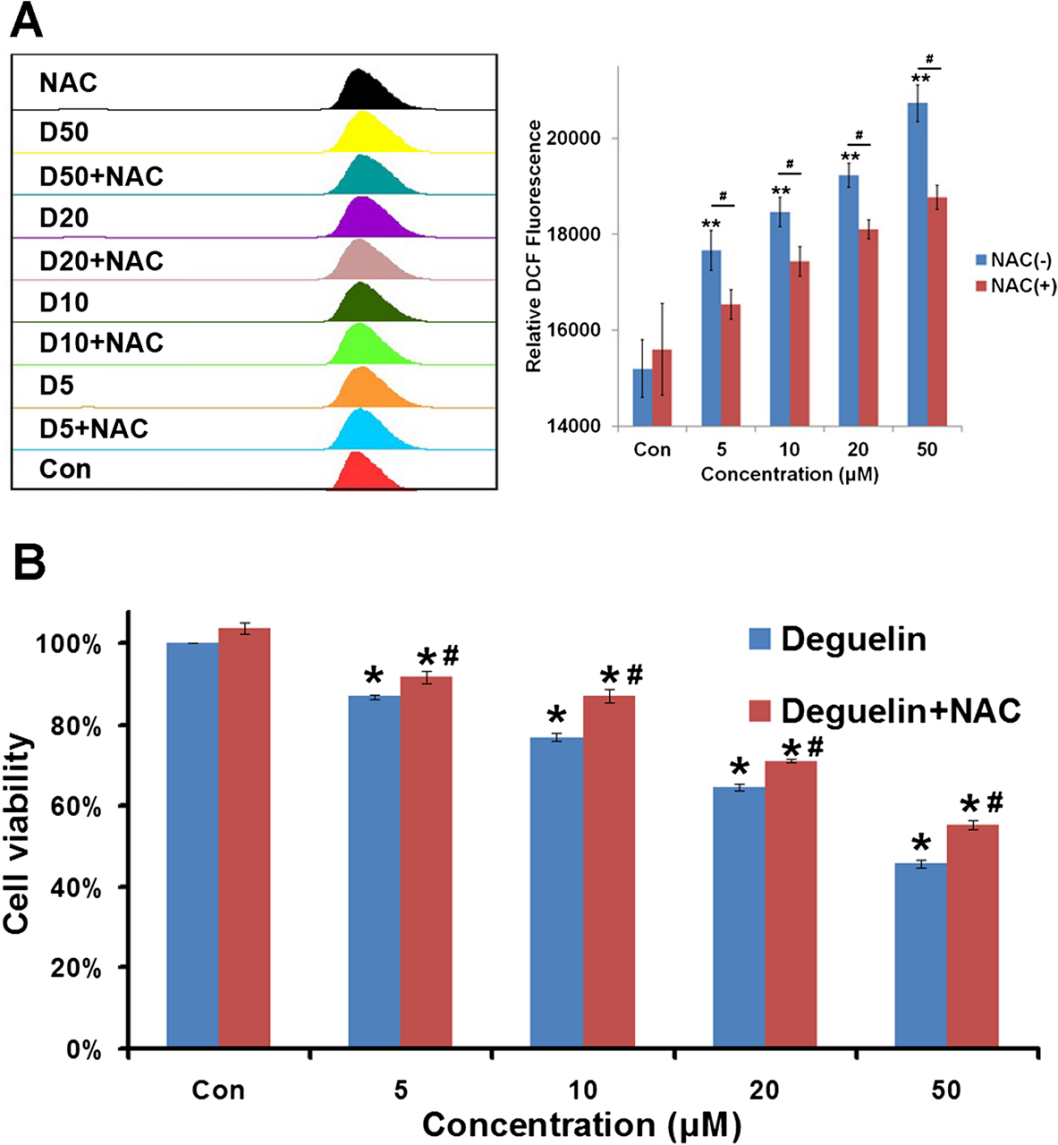
**Intracellular ROS induction and in vitro cytotoxicity of Deguelin. A**: Quantitative intracellular DCF fluorescence intensity of NCI-1975 cells induced by Deguelin with or without the presence of NAC. # represents p < 0.05 vs the corresponding dose between cells without and with the treatment of NAC. ** represents p < 0.01 vs the control cells. Data are presented as mean ± SD (n = 3). **B**: Cell cytotoxicity test of Deguelin with or without the presence of NAC in NCI-1975 cells after 24 h incubation. # represents p < 0.05 vs the corresponding dose between cells without and with the treatment of NAC. * represents p < 0.05 vs the control cells. Data are represented as Mean ± SD (n = 3).

Intracellular ROS levels were evaluated by FACS with H_2_DCF-DA as a fluorescent probe. Shown in Figure 1A, Deguelin induced the generation of ROS in a dose-dependent manner, which was partially blocked by co-treatment with NAC. For example, a nearly 10% reduction in ROS levels was observed in the cells receiving 50 μM Deguelin plus NAC in comparison to the corresponding dose of Deguelin alone. The reversal effect of NAC on ROS production seems to be weak, but statistical analyasis showed that there was a significant difference in ROS levels between Deguelin alone and combined delivery of Deguelin and NAC (p < 0.05).

### Deguelin induces the apoptosis of NCI-1975 cells in a ROS-dependent manner

It is shown in Figure 1B by the MTT assay that cell viability decreased with the concentration of Deguelin increasing. Moreover, the dose dependent inhibitory effect of Deguelin on NCI-1975 cells was partially but statistically significantly reversed by the antioxidant NAC. As calculated from the bar graph, the IC50 value of Deguelin was 42.6 μM while that of Deguelin with the co-treatment of NAC was 54.7 μM. There was a significant difference between the cytoxicity of Deguelin with and without the co-incubation of NAC (p < 0.05). In addition, we tested the cytotoxicity of Deguelin on another kind of lung cancer line NCI-H446. It showed the same trend as on NCI-1975 with a slightly lower IC50 at about 38.3 μM (Suppl. Figure 1).

**Figure 2:**
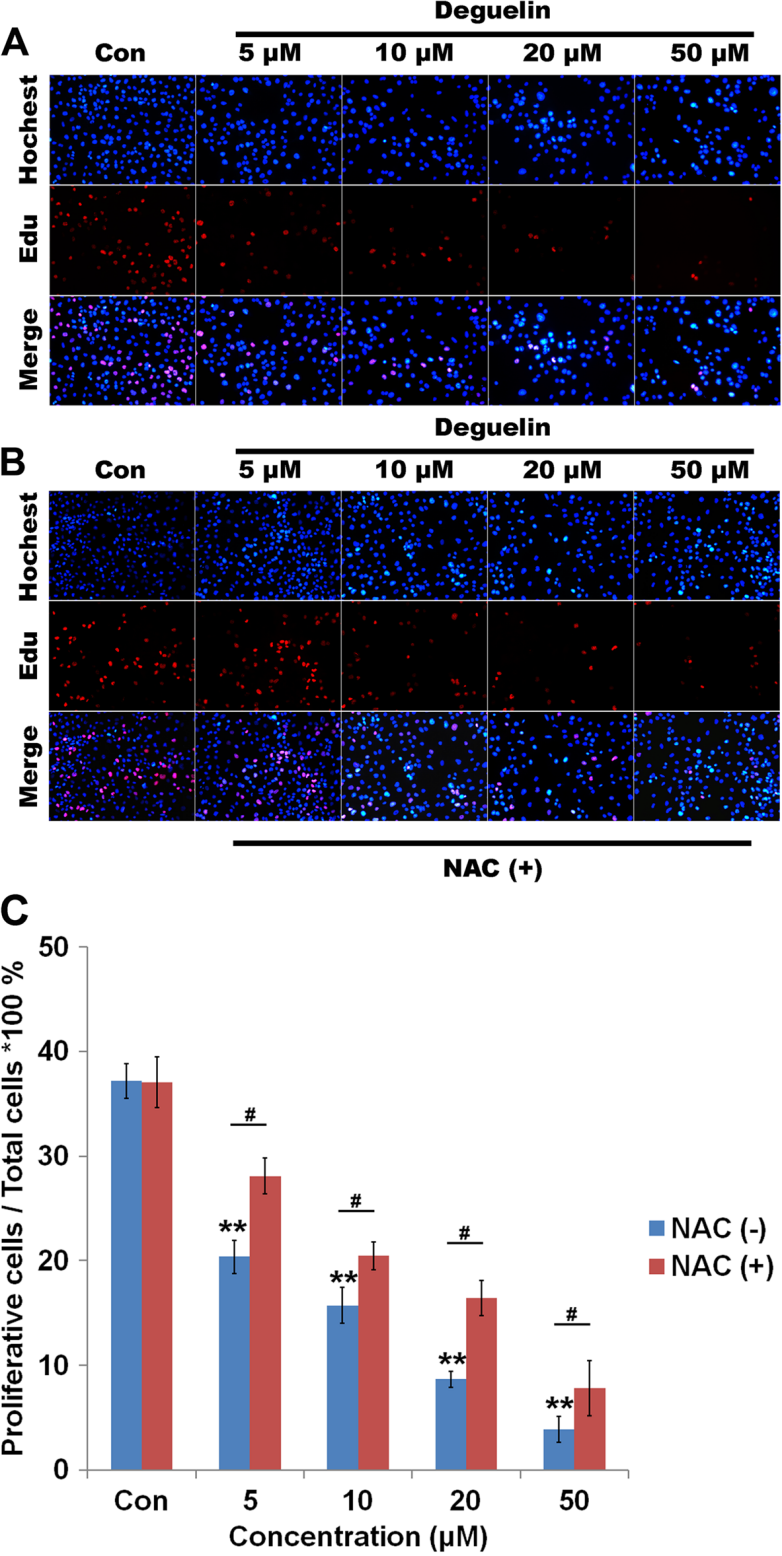
**Influence of Deguelin on the cell proliferation. A**: Hoechest and Edu dual staining of NCI-1975 cells under the treatment of a series of Deguelin with or without the presence of NAC. A: Image of dual staining of cells treated by Deguelin only. **B**: Image of dual staining of cells treated by Deguelin with the presence of NAC (500 μM). **C**: Quantification of proliferative cells under the treatment of Deguelin with or without the presence of NAC. # represents p < 0.05 vs the corresponding dose between cells without and with the treatment of NAC. ** represents p < 0.01 vs the control cells. Data are presented as mean ± SD (n = 3).

The anti-growth effect of Deguelin was also measured by double staining with Edu and Hoechest 33432, which simultaneously manifested the proliferating (Red staining by Edu) and apoptotic (Bright Blue staining by Hoechest 33432) cells, respectively. As shown from Figure 2A, there was an obvious dose-dependent decrease in the number of proliferating cells as well as a substantial increase in apoptotic cells when cells were treated by a series of doses of Deguelin. Most importantly, the cytotoxicity of Deguelin was attenuated by the co-administration of NAC, which is consistent with the results from cyotoxicity studies.

The quantitative analysis of cell proliferation and apoptosis (Figure 2B) also indicated that Deguelin dose-dependently inhibited the proliferation and induced the apoptosis of NCI-1975 cells, which could be counteracted by the co-administration of NAC. Statistical analysis revealed that co-treatment of NAC with Deguelin significantly increase the percentage of proliferating cells compared to Deguelin treatment alone (p < 0.05).

**Figure 3:**
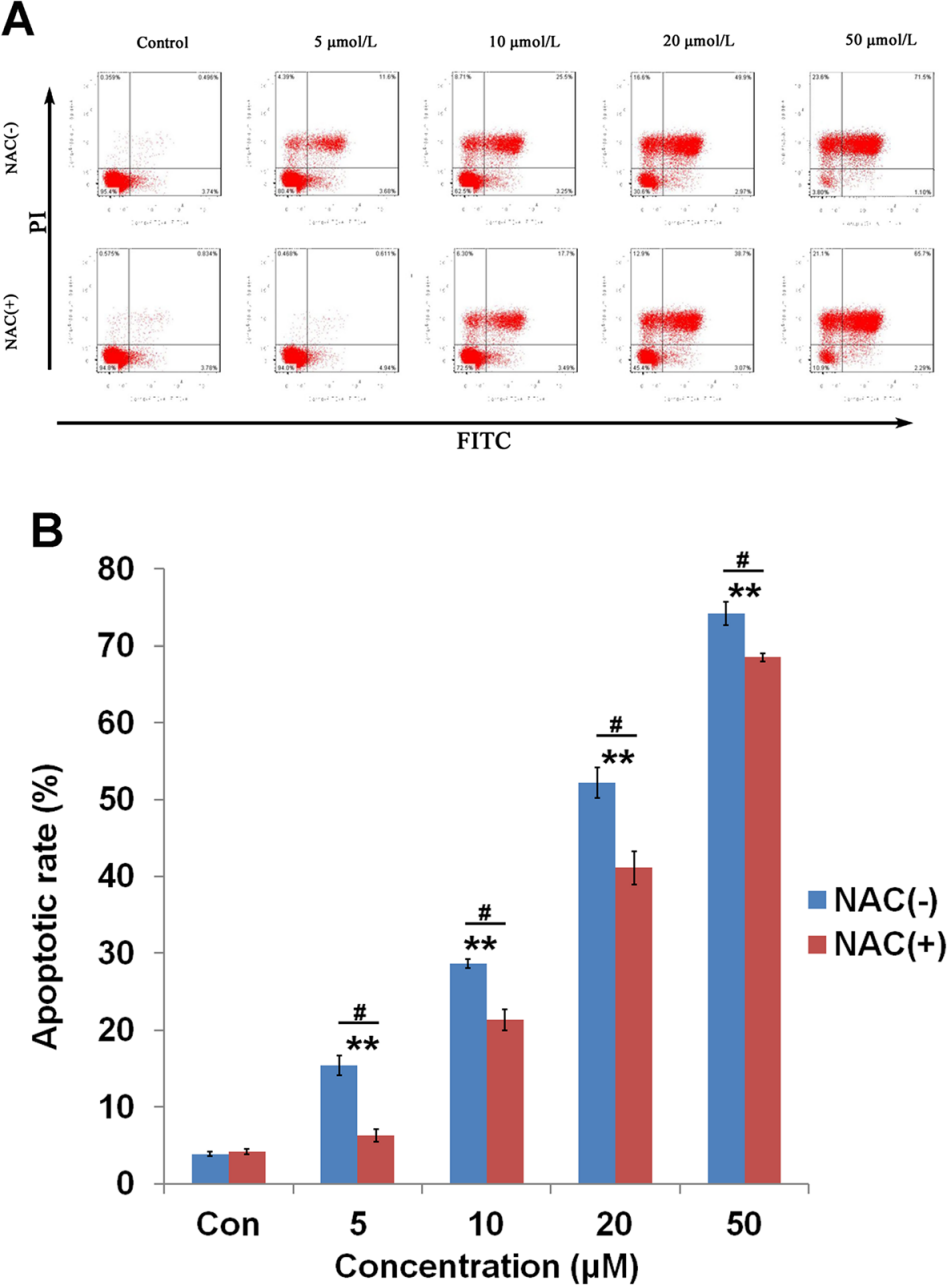
**Apoptosis induced by Deguelin. A**: Apoptosis rates induced by Deguelin with or without the co-treatment of NAC by FACS. **B**: Quantification of apoptotic cells induced by Deguelin with or without the co-treatment of NAC. # represents p < 0.05 vs the corresponding dose between cells without and with the treatment of NAC. ** represents p < 0.01 vs the control cells. Data are presented as mean ± SD (n = 3).

In addition, cellular apoptosis induced by Deguelin was also measured by FACS quantitatively, which showed the same trend observed in both cytotoxicity and proliferation assays (Figure 3A). Increasing the concentration of Deguelin led to an elevation of cellular apoptosis, which was significantly reversed by co-treatment with NAC (p < 0.05 vs Deguelin alone) (Figure 3B). Moreover, it is interesting that Deguelin increases the number of late apoptotic (necrotic) cells rather than early apoptotic cells. For example, late apoptosis increased significantly from less than 20% to more than 60% when the concentration of Deguelin increased from 5 μM to 50 μM.

### The apoptosis induced by Deguelin is mediated through a ROS-driven Akt pathway

**Figure 4:**
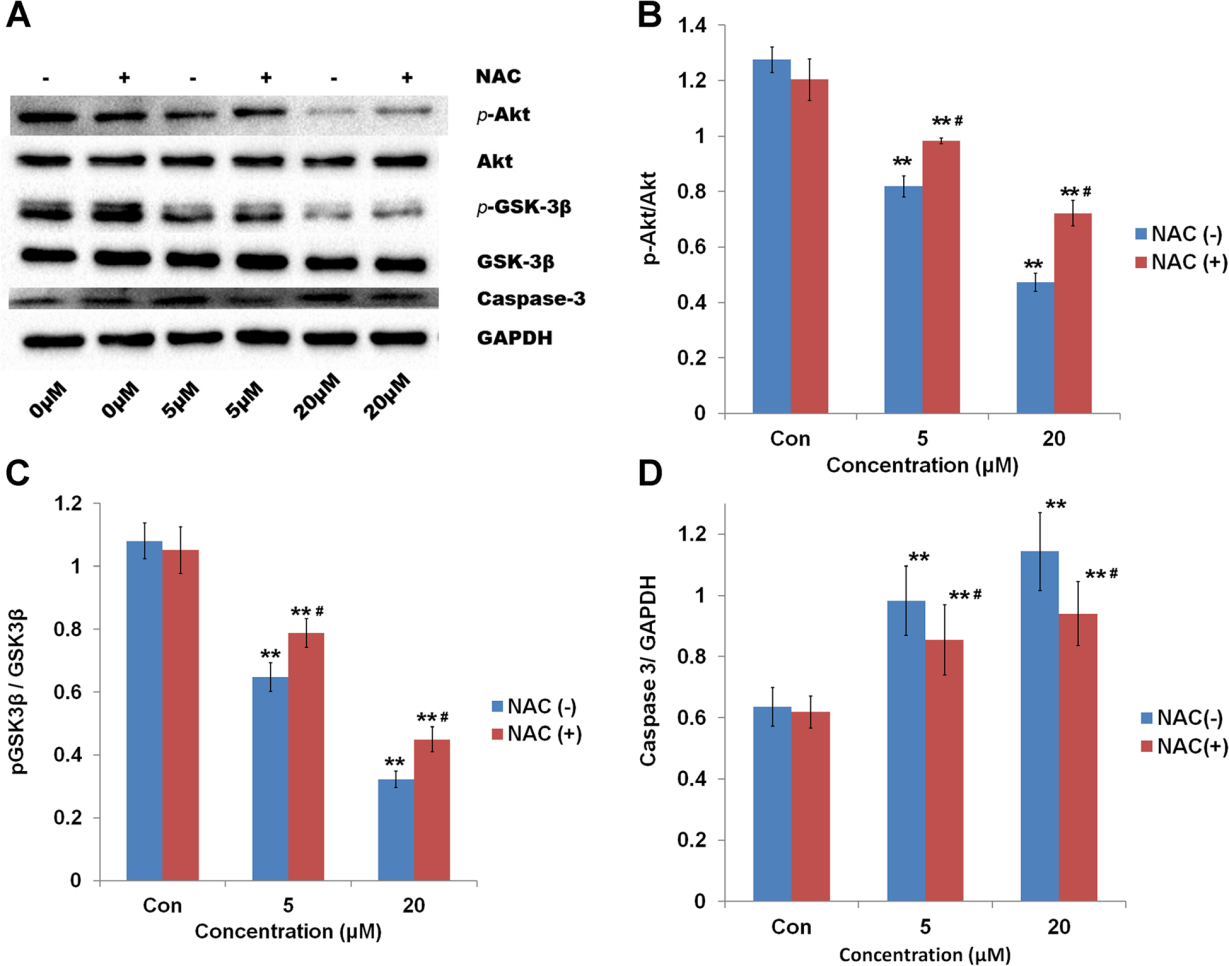
**Relative protein expression under the treatment of Deguelin. A**: Gel image of Western blot analysis showing the related protein expression in NCI-1975 cells exposed to Deguelin (5 and 20 μM) with or without the presence of NAC. **B**: Bar graph representing the semi-quantification of gel image (p-Akt/Akt) normalizing the band with the GAPDH control. # represents p < 0.05 vs the corresponding dose between cells without and with the treatment of NAC. ** represents p < 0.01 vs the control cells. Data are presented as mean ± SD (n = 3). **C**: Bar graph representing the semi-quantification of gel image (p-GSK3β/GSK3β) normalizing the band with the GAPDH control. # represents p < 0.05 vs the corresponding dose between cells without and with the treatment of NAC. ** represents p < 0.01 vs the control cells. Data are presented as mean ± SD (n = 3). **D**: Bar graph representing the semi-quantification of gel image (Caspase-3) normalizing the band with the GAPDH control. # represents p < 0.05 vs the corresponding dose between cells without and with the treatment of NAC. ** represents p < 0.01 vs the control cells. Data are presented as mean ± SD (n = 3).

Western blot analysis revealed that Deguelin could greatly ameliorated the expression of p-Akt and p-GSK3β and subsequently activated the caspase-3 pathway (Figure 4A). Moreover, NAC significantly reduced the suppressive effect of Deguelin on protein expression. In addition, semi-quantitative analysis of the bands showed a significant decrease in the expression of p-Akt and p-GSK3β as the concentration of Deguelin increased, regardless of co-treatment with NAC (p < 0.05) (Figure 4B & C). Most importantly, significant differences were noted in the expression of p-Akt and p-GSK3β between the cells exposed to single Deguelin treatment and combination treatment with NAC. In comparison to control cells, the expression of p-Akt was significantly reduced by more than 30% in the cells treated with 5 μM Deguelin. Meanwhile, co-treatment with NAC restored p-Akt expression by nearly 20% (Figure 4B & C).

Additionally, the expression of cleaved Caspase-3, an apoptotic indicator, increased dramatically during the cellular exposure to two different doses of Deguelin (Figure 4A). Co-treatment with NAC resulted in more than 20% reduction in the elevated levels of cleaved Caspase-3 observed in Deguelin-only treated cells (Figure 4D).

## Discussion

As previously reported, tumor cells are vulnerable to the induced ROS generation due to the insufficiency of antioxidant mechanisms [24,27]. Intracellular ROS accumulation by some chemotherapeutics is one of the most important strategies of inducing the apoptosis of cancer cells [28,29]. On the contrary, strengthening the antioxidant capacity by introducing small ROS scavenging molecules, such as NAC, will reduce the potential of the drug to induce apoptosis. NAC is the most common used discriminator to verify if cell death is associated with ROS. Some studies show that NAC exhibits a concentration-dependent inhibition on ROS induction [30]. However, other studies demonstrate that NAC is a weak reducing agent and a poor antioxidant compared with glutathione (reduced form) (GSH) [31,32].

In our previous studies, we found that several herbal medicines, such as Tetrandrine, Resveratrol, Ursolic acid, etc., can influence the redox state of cancer cells by generating intracellular ROS, thereby contributing to cellular apoptosis, which can be partially reversed by antioxidants, such as NAC, Vitamin E [18,33-35]. Few studies to date focus on the effects of Deguelin on the cellular redox state. Only one study reported that Deguelin is capable of rapidly diminishing the oxygen consumption by interfering with Complex I to trigger mitochondrial permeability transition and result in an oxidative stress with excess ROS, thereby leading to the apoptosis [36].

In the current study, Deguelin exerted an oxidative stress on NCI-1975 cells by elevating the intracellular level of ROS, which was partially reversed by co-treatment with the antioxidant NAC. In the cytotoxicitity test, Deguelin shows a dose-dependent inhibitory effect on the proliferation of NCI-1975 cells, while co-administration of NAC resulted in partial rescue of some cells with an increased survival rate. Further analysis of the intracellular ROS levels reveals that Deguelin augments the generation of intracellular ROS in a dose dependent manner. Similarly, antioxidant NAC co-treatment partially blocks the stimulating effect of Deguelin in ROS generation. Though there was only 10% reduction of ROS production under the treatment of NAC, it still generated statistically significant reversal effect against the cytotoxicity of Deguelin. It means that ROS elevation is significant to the cytotoxicity of Deguelin. A subtle alteration of ROS levels can lead to substantial change in the cytotoxicity of Deguelin. Therefore, like other herbal medicines, Deguelin possesses the ability to induce cellular oxidative stress, offering a promising potential therapeutic for lung cancer therapy.

Previous studies have demonstrated that Deguelin could induce both early and late apoptosis at certain concentrations [4]. However, our results provided another possibility of Deguelin’s effect. FCAS analysis showed that Deguelin induced more late apoptosis (necrosis) than early apoptosis, which is a very interesting phenomenon. Literature search in the database showed that in addition to apoptosis, Deguelin could induce autophagay, another kind of cell death, to inhibit the growth of cancer cells [37]. Therefore, it is possible that the cytotoxicity of Deguelin not only involves cell apoptosis but also other kinds of cell death, such as autophagy. Further studies needs to be performed to clarify this phenomenon.

PI3k/Akt, one of the main mediators of survival signals preventing cells from undergoing apoptosis, tends to be a potential target for cancer therapy [38,39]. Previous studies focused on the antitumor effect of PI3k/Akt inhibitors, such as LY294002 and wortmannin, etc. [40,41]. However, the toxicity and poor solubility of these inhibitors still pose a major barrier for further application. Previous studies reported that Deguelin, a naturally occurring herbal medicine, inhibits the phosphorylation of Akt and induces apoptosis in a series of cancers, such as lung, head and neck, gastric cancer etc. [4,42]. In addition, the current findings are also valuable to overcome the emerging resistance to chemotherapeutics in cancer cells. Earlier studies have demonstrated that drug resistance is closely related to the activation of PI3k/Akt pathway [43,44]. Thus, Deguelin, as a natural PI3k/Akt inhibitor, seems to be a novel antitumor drug targeting the Akt pathway.

Moreover, evidence from earlier studies show that there seems to be a hierarchy in the relationship between ROS and Akt, in which ROS tends to function as an upstream signal for Akt [24,45,46]. In the current study, we demonstrated for the first time that both the activity of PI3k/Akt pathway and the cellular redox state are closely related to the pharmacological effect of Deguelin. The antitumor effect of Deguelin is mediated through the ROS-driven dephosphrylation of Akt, which indicates the potential of Deguelin in the targeted therapy of ROS dependent PI3k/Akt pathway. However, ROS detection by FCAS showed that only 10% of ROS production was inhibited by NAC. Though there is a significant difference between the presence and absence of NAC, this partial reversal effect of NAC still means ROS plays a key role in Deguelin’s anticancer effect.

Future studies will be focused on the chemosensitization effect of Deguelin. Previous studies show that accumulation of ROS is a crucial step for Paclitaxel-induced cell apoptosis, and that cellular antioxidant capacity plays a key role in the chemoresistance of Paclitaxel [28]. Since Deguelin is able to induce oxidative stress, it is reasonable to hypothesize that Deguelin may sensitize cancer cells to Paclitaxel through intracellular induction of ROS, which can shed light on the novel chemosensitization strategy in cancer therapy.

## Conclusions

We report in the current study that Deguelin, a natural retinoid isolated from some plant species, effectively inhibits the growth and induces the apoptosis of NSCLC NCI-1975 cells through the elevation of intracellular ROS levels, and following inactivation of downstream phospho-Akt protein.

## Materials and methods

### Reagents

Deguelin, 2,7-dichlorodihydrofluorescin diacetate (H_2_DCF-DA), N-acetylcysteine (NAC), dimethyl sulfoxide (DMSO) and 3-(4,5-dimethylthiazol-2-yl)-2,5-diphenylformazan (MTT) were purchased from Sigma Chemical Co. (St. Louis, MO, USA). Stock solutions of H_2_DCF-DA and NAC were made in DMSO and sterilized by passage through a 0.22 μm pore size filter (Immobilon, Millipore Corp., Bedford, MA, USA), diluted with culture media before use. RPMI-1640 medium, fetal bovine serum (FBS) and penicillin/streptomycin were purchased from Gibco BRL (Grand Island, NY, USA). Annexin V-fluorescein isothiocyanate (FITC) kit was purchased from Bender MedSystems (Vienna, Austria). Other reagents were of analytic grade and obtained from Nanjing Chemical Reagent Co. (Nanjing, China), unless otherwise described.

### Cell culture

Human non small cell lung cancer cell line NCI-1975 was obtained from the Institute of Biochemistry and Cell Biology, Chinese Academy of Sciences (Shanghai, China). The cells were cultured in RPMI-1640 medium supplemented with 10% FBS, 100 units/ml penicillin G, and 100 μg/ml streptomycin at 37°C in a humidified incubator with 5% CO_2_.

### Methyl thiazolyl tetrazolium (MTT) assay

NCI-1975 cells were seeded into 96-well plates at a density of 5 × 10^3^ cells per well and incubated overnight in 10% FBS RPMI 1640 medium. The cells were then treated with a series of Deguelin (5, 10, 20, 50 μM) in serum-free conditions for 24, 36 or 48 h, with or without the co-treatment of NAC (500 μM). The cell proliferation was determined by the MTT assay as described in our previous study [18,47]. The 50% inhibitory concentration (IC_50_) was calculated using the dose–response curve.

### Edu staining for cell proliferation

NCI-1975 cells were treated with Deguelin (5, 10, 20, 50 μM) with or without the co-treatment of NAC (500 μM) and then washed once with PBS. The cells were stained with 300 μl 50 μM Edu solution for 2 hours according to the protocol. Cells were then stained with the DNA-specific fluorochrome Hoechst 33342. After incubation in the dark at 37°C for 15 min, the cells were washed with PBS, air-dried, mounted with 90% (v/v) glycerol, and examined using a fluorescence microscope with an excitation/emission wavelength of 350/550 nm (Olympus, Japan). Proliferative cells were identified by counting the red cells in ten different fields.

### Annexin V/propidium iodide staining

NCI-1975 cells were seeded in a cell culture dish and exposed to a series of Deguelin (5, 10, 20, 50 μM) with or without the co-treatment of NAC (500 μM) for 24 h. Apoptosis in NCI-1975 cells was evaluated by annexin V-FITC and propidium iodide (PI) staining using an Annexin V-FITC kit according to the manufacturer’s protocol. After incubation in the dark at room temperature for 15 min, the cells were immediately analyzed by FACScan flow cytometer (Becton Dickinson, CA, USA).

### Detection of intracellular ROS production

Intracellular ROS generation was detected using H_2_DCF-DA as a fluorescent probe, which is a non-polar compound that is converted into a non-fluorescent polar derivative (H_2_DCF) by cellular esterases after incorporation into cells. Membrane-impermeable H_2_DCF is rapidly oxidized to highly fluorescent 2′,7′-dichlorofluorescein (DCF) in the presence of intracellular ROS. NCI-1975 cells were plated at a density of 1 × 10^6–7^ in 6-well plates, allowed to attach overnight, and exposed to Deguelin (5, 10, 20, 50 μM) with or without the co-treatment of NAC (500 μM) for 24 h, followed by staining with 10 μM H_2_DCF-DA for an additional 30 min, rinsed with PBS. In another experiment, cells were pre-treated with NAC at 400 μM for 2 h and then treated with Deguelin. The fluorescent DCF intensity was quantified using a FACScan flow cytometer (Becton Dickinson, CA, USA).

### Western-blot analysis

NCI-1975 cells were grown to sub-confluence in 60 mm dishes and thereafter cultured in serum-free medium for 24 h. The cells were then treated with Deguelin (5 and 20 μM) with or without the co-treatment of NAC (500 μM) for 24 h. The proteins were extracted from cells and western blot analyses were performed as described in our previous reports. Antibodies used include rabbit anti-phospho-Akt (Ser 473), anti-Akt, anti-pGSK3β, anti-Caspase-3 (Cell Signaling Technology, Danvers, MA, USA); and mouse anti-β-actin (Sigma). Goat anti-rabbit and anti-mouse IgG horseradish peroxidase (HRP)-conjugated secondary antibodies were purchased from the Jackson ImmunoResearch Laboratories (West Grove, PA, USA). Bands were quantified using densitometric image analysis software (Quantity One, Bio-Rad, Hercules, CA, USA). The relative expression of phospho-Akt (p-Akt) was normalized to total Akt (t-Akt) level.

### Statistical analysis

The data is shown as the mean ± standard deviation (SD), and was analyzed either by Student’s *t* test or by one-way analysis of variance (ANOVA) (SPSS version 11.0; SPSS Inc., Chicago, IL, USA). A probability value of less than 0.05 was considered statistically significant.
